# Supplementary material for: Development of Thermosensitive and Mucoadhesive Hydrogel for Buccal Delivery of (S)-Ketamine
Source: Pharmaceutics. 2022 Sep 24;14(10):2039. doi: 10.3390/pharmaceutics14102039 (PMC9608784; doi:10.3390/pharmaceutics14102039)
Supplement: Supplementary file 1 [file pharmaceutics-14-02039-s001.zip › pharmaceutics-1899386-supplementary.pdf]

Table S1. Storage moduli of polymer solutions (G'$_p$) and polymer-mucin mixtures (G'$_t$) measured at 1 Hz frequency and calculated interaction parameter (ΔG'). Data were reported as the mean ± SD (n = 3).

Samples	G'$_p$ (Pa)	G'$_t$ (Pa)	ΔG' (Pa)
P407	6700 ± 400	6400 ± 200	-400 ± 200
P407/PRO_{10}	5500 ± 300	4100 ± 500	-1400 ± 700
P407/PRO_{15}	6200 ± 100	3900 ± 300	-2200 ± 200
P407/PRO_{20}	6000 ± 100	2700 ± 500	-3300 ± 500
P407/KEL_{10}	6000 ± 200	4000 ± 200	-2000 ± 300
P407/KEL_{15}	5400 ± 300	3600 ± 500	-1900 ± 400
P407/KEL_{20}	4100 ± 300	2200 ± 600	-2000 ± 800
P407/MAN_{10}	6200 ± 400	4300 ± 100	-1900 ± 500
P407/MAN_{15}	5500 ± 200	3000 ± 700	-2600 ± 800
P407/MAN_{20}	5100 ± 300	3000 ± 500	-2100 ± 800

P407: poloxamer 407; PRO: Protanal LF 10/60; KEL: Keltone LVCR; MAN: Manucol DH.

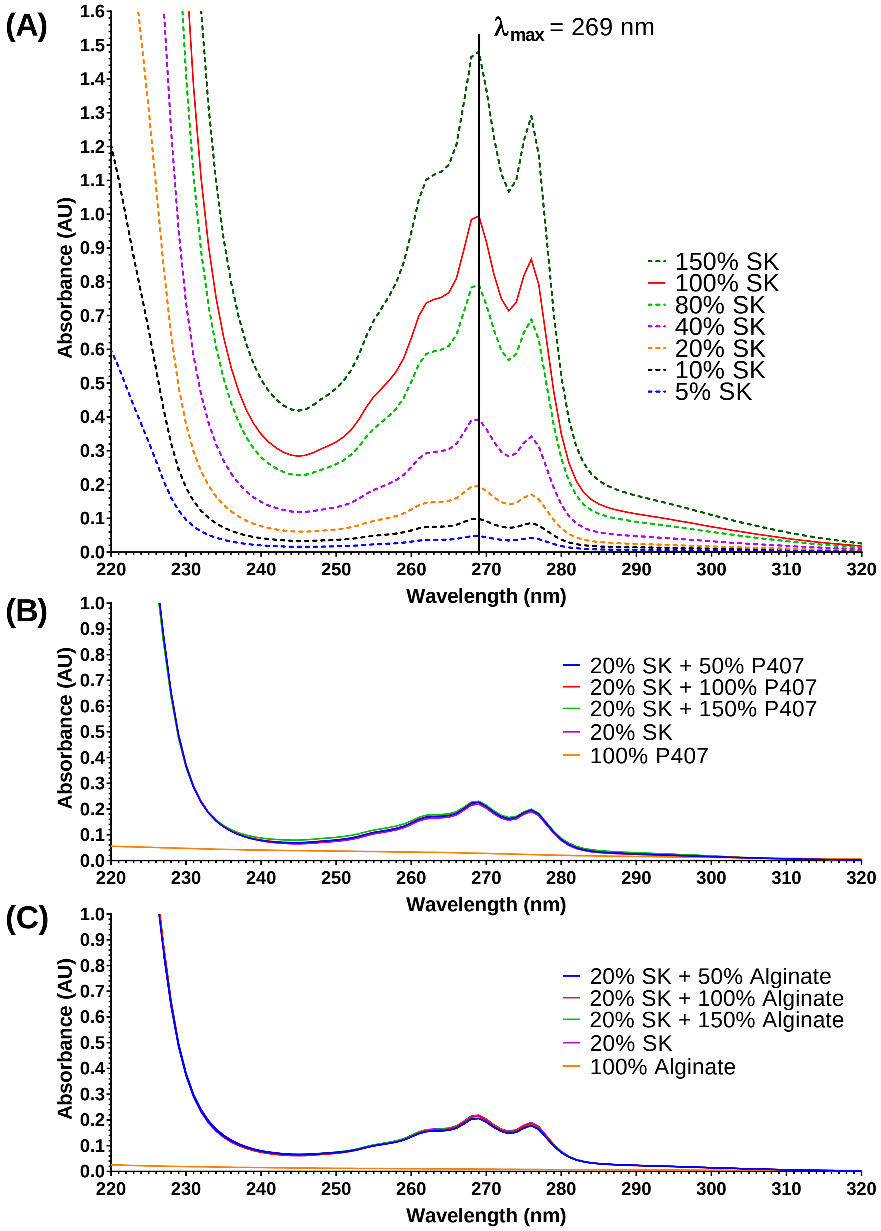

Figure S1. (A) UV spectra of (*S*)-ketamine hydrochloride (SK) at various concentrations expressed as percentages of the maximal concentration awaited during dissolution testing ("100% SK" = 0.52 mg/mL). UV spectra of (B) P407, SK and SK/P407 mixtures, (C) alginate, SK and SK/alginate mixtures. Different proportions of excipients were studied (100% corresponding to the total release of excipient) with a constant concentration "20% SK" (0.10 mg/mL).
